# Perceptions of the US National Tobacco Quitline Among Adolescents and Adults: A Qualitative Study, 2012–2013

**DOI:** 10.5888/pcd12.150139

**Published:** 2015-08-20

**Authors:** Erika A. Waters, Amy McQueen, Charlene A. Caburnay, Sonia Boyum, Vetta L. Sanders Thompson, Kimberly A. Kaphingst, Matthew W. Kreuter

**Affiliations:** Author Affiliations: Amy McQueen, Washington University School of Medicine, St. Louis, Missouri; Charlene A. Caburnay, Sonia Boyum, Vetta L. Sanders Thompson, Matthew W. Kreuter, Washington University in St. Louis, St. Louis, Missouri; Kimberly A. Kaphingst, University of Utah, Salt Lake City, Utah.

## Abstract

**Introduction:**

Tobacco quitlines are critical components of comprehensive tobacco control programs. However, use of the US National Tobacco Quitline (1-800-QUIT-NOW) is low. Promoting quitlines on cigarette warning labels may increase call volume and smoking cessation rates but only if smokers are aware of, and receptive to, quitline services.

**Methods:**

We conducted qualitative interviews with a diverse subset (n = 159) of adolescent (14–17 y) and adult (≥18 y) participants of a larger quantitative survey about graphic cigarette warning labels (N = 1,590). A convenience sample was recruited from schools and community organizations in 6 states. Interviews lasted 30 to 45 minutes and included questions to assess basic knowledge and perceptions of the quitline number printed on the warning labels. Data were analyzed using content analysis.

**Results:**

Four themes were identified: available services, caller characteristics, quitline service provider characteristics, and logistics. Participants were generally knowledgeable about quitline services, including the provision of telephone-based counseling. However, some adolescents believed that quitlines provide referrals to “rehab.” Quitline callers are perceived as highly motivated — even desperate — to quit. Few smokers were interested in calling the quitline, but some indicated that they might call if they were unable to quit independently. It was generally recognized that quitline services are or should be free, confidential, and operated by governmental or nonprofit agencies, possibly using tobacco settlement funds.

**Conclusion:**

Future marketing efforts should raise awareness of the nature and benefits of quitline services to increase use of these services and, consequently, reduce tobacco use, improve public health, and reduce tobacco-related health disparities.

## Introduction

Tobacco use is the leading cause of preventable morbidity and mortality worldwide (1). Tobacco quitlines, which provide tobacco users free telephone counseling, nicotine replacement therapy (NRT), and other cessation services, are important components of comprehensive tobacco control programs (2) and can increase 6-month abstinence rates dramatically (3–7). However, limitations in awareness and use reduce the reach of quitlines. In 2009–2010, only 54% of current smokers and 34% of former smokers in the United States reported being aware of telephone-based smoking cessation services (8). Furthermore, only 7.8% of smokers who were aware of quitlines and had tried to quit in the previous year contacted a quitline for assistance.

There have been extensive efforts to expand the awareness and use of quitlines among smokers. Mass media campaigns are effective in increasing call volumes and smoking cessation rates (9–13). Nevertheless, such approaches are short-lived and do not reach all smokers (13–15). Additional efforts are needed to promote quitline awareness and use, particularly among populations that have less success in quitting (eg, racial/ethnic minorities).

Prior studies demonstrated that quitlines may be underused due to barriers such as not perceiving the need for assistance and being concerned about the stigma associated with seeking help (16,17). However, participants in those studies were predominantly white and highly educated adults. The objective of this study was to better understand basic knowledge and perceptions of quitlines among populations that have a disproportionate share of tobacco-related morbidity and mortality and are underrepresented in quitline research. The results will inform the development and implementation of future media campaigns by identifying potential targets for messaging.

## Methods

All procedures and materials were approved by the Washington University institutional review board. The data reported here represent a subset of data collected for the purpose of understanding public reactions to 9 graphic cigarette warning labels proposed by the US Food and Drug Administration (18). The labels included a photo or drawing, warning text, and the telephone number for the US national tobacco quitline (1-800-QUIT-NOW). Data collection activities included a quantitative survey (N = 1,590) and, for a subset of respondents, an in-person, one-on-one, semistructured qualitative interview (n = 159). The primary aim of the qualitative interview was to assess conceptual understanding of the warning label messages. This study, which reports basic knowledge and perceptions of the national tobacco quitline, is a secondary analysis of the qualitative interview data.

### Sample characteristics and setting

We recruited participants by using targeted recruitment strategies based on existing contacts with community and governmental organizations and personal outreach by leaders and members of underrepresented groups. We enrolled a convenience sample of participants aged 13 years or older from 6 population subgroups with high rates of smoking-related morbidity and mortality: low-income Americans, African Americans, American Indians, US military personnel, rural residents, and blue-collar workers (19–23). There were no other inclusion or exclusion criteria. Recruitment occurred at schools, trade unions, and tribal, community, and military (USO) organizations and events.

We attempted to recruit 10% of the survey participants (n = 160) for the qualitative interviews (ie, 10 from each of the 6 population subgroups from each of 3 age groups [13–17 y, 18–24 y, and ≥25 y]; there were no adolescent military or blue-collar participants). Each participant represented only 1 subgroup, but subgroups were not mutually exclusive (eg, African American blue-collar workers). Interview participants were selected according to practical considerations (eg, quotas, interviewer availability). Due to organizational constraints, we recruited only 8 of the planned 20 members of the military. One participant’s data were unusable because of a data collection error, resulting in 159 usable qualitative interviews. Interviews were conducted from June 2012 through March 2013 at 36 recruitment locations in 6 states: Florida (3 locations, n = 5), Iowa (4 locations, n = 6), Illinois (2 locations, n = 11), Kansas (2 locations, n = 9), Missouri (22 locations, n = 108), and New York (3 locations, n = 20).

### Sociodemographics and smoking status

In the quantitative survey, participants used standard measures to indicate their age, sex, race/ethnicity, educational attainment (adults) or grade level (adolescents), and income (adults) or receipt of free or reduced-price school lunches (adolescents). Adults earning less than $25,000 per year and adolescents reporting free or reduced-price lunches were considered low income. To determine whether participants were rural residents, we applied the RUCA (rural–urban commuting areas) taxonomy to recruitment locations; a rural area was defined as having a RUCA code of 4 or more (24). Standard smoking-related variables were lifetime use of cigarettes (≥100 vs <100) and current use of cigarettes (every day, some days, not at all). Adolescents who reported smoking on at least 1 of the past 30 days were considered smokers (25). Adults were considered smokers if they currently smoked cigarettes every day or some days and had smoked more than 100 cigarettes in their lifetime. Adults who had smoked at least 100 cigarettes but not at all in the past 30 days were considered former smokers.

Quitlines are likely less salient and relevant to nonsmokers than smokers. Nevertheless, nonsmokers were included because their knowledge and perceptions contribute to social norms and stigma and therefore may affect whether they recommend the quitline to family or friends who smoke (26).

### Study procedures

Trained interviewers conducted the qualitative interviews in private rooms at the recruitment location. Interviews were conducted immediately after survey administration or were scheduled for a later date. Interviews lasted 30 to 45 minutes and were audiorecorded. Participants received a $20 gift card.

Consistent with the overall study’s primary goal, most of the qualitative interview questions assessed conceptual understanding of, and reactions to, the message conveyed by graphic images and text warning messages. A small number of questions assessed basic knowledge about the function of a tobacco quitline and perceptions of callers. These questions were tailored to the participant’s age and smoking status to ensure that they were valid for all participants. Examples of questions are “What do you think the [quitline] number is all about?” “What do you think would happen if you [smokers]/someone [nonsmokers] called that number?” “Who would you [smokers]/the caller [nonsmokers] be talking to?” and “Who would/would not call this number?”

Several quality assurance strategies were undertaken. First, all interviewers received didactic and hands-on training in qualitative interviewing skills, including role-playing exercises. Second, an interview guide was developed that identified the topics of interest and provided interviewers with suggested wording for questions and probes. Third, transcripts were reviewed periodically to ensure adequate performance and determine whether the interview guide needed modifications. Fourth, data quality was monitored after each recruitment event.

### Analysis

Audio recordings were transcribed verbatim. Because the responses were brief, we conducted qualitative content analysis (27). Analysis focused on the manifest content or stated meaning of responses at the participant level, with the goal of making the results relevant to practitioners and policy makers (28,29). E.A.W. and A.M. independently reviewed all responses. Using an iterative strategy, similar responses were grouped into codes. E.A.W. and A.M. met periodically to discuss the codes and achieve consensus. Similar codes were grouped into themes. Sample quotes were identified that illustrated common and potentially unique perceptions within each code. E.A.W. and A.M. independently explored responses for any differences by age, race/ethnicity, rural residency, and smoking status. All authors participated in the final interpretation of the analysis. Atlas.ti (Scientific Software Development GmbH) and Microsoft Excel (Microsoft Corp) were used to organize the data for analysis.

## Results

The sample was diverse in age, race, and socioeconomic status (Table 1). Most adolescents were nonsmokers. There were more females, racial minorities, and people with low income among adolescents than adults.

**Table 1:** Sociodemographic Characteristics of Interview Participants (N = 159), Study on Perceptions of the US National Tobacco Quitline Among Adolescents and Adults, 2012–2013^a^^,^ b

Characteristic	Age 13–17 Years (N = 42)	Age 18–24 Years (N = 60)	Age ≥25 Years (N = 57)
**Age, mean (SD), y**	15.0 (1.4)	21.4 (2.1)	40.8 (10.4)
**Sex**			
Male	17 (40.5)	36 (60.0)	33 (57.9)
Female	25 (59.5)	24 (40.0)	24 (42.1)
**Education**			
<High school	32 (76.2)	5 (8.3)	10 (17.5)
High school/GED	7 (16.7)	16 (26.7)	11 (19.3)
>High school	0 (0)	38 (63.3)	36 (63.2)
Missing	3 (7.1)	1 (1.7)	NA
**Smoking status**			
Nonsmokers	40 (95.2)	5 (8.3)	15 (26.3)
Former smokers	NA	30 (50.0)	14 (24.6)
Smokers	2 (4.8)	25 (41.7)	28 (49.1)
**Race**			
White	11 (26.2)	26 (43.3)	30 (52.6)
African American^c^	16 (38.1)	15 (25.0)	12 (21.1)
American Indian^c^	11 (26.2)	13 (21.7)	10 (17.5)
Other	4 (9.5)	6 (10.0)	5 (8.8)
**Other**			
Low income^c^	23 (NA)	26 (NA)	24 (NA)
Rural^c^	19 (NA)	16 (NA)	21 (NA)
Military^c^	NA	7 (NA)	1 (NA)
Blue collar^c^	NA	10 (NA)	10 (NA)

Abbreviations: GED, general educational development; NA, not applicable.

a All values are number (percentage) unless otherwise indicated.

b In-person interviews were conducted in private rooms at 36 recruitment locations in 6 states: Florida, Iowa, Illinois, Kansas, Missouri, and New York.

c Subgroups for which we attempted to recruit at least 10 participants each; groups are not mutually exclusive, so percentages do not sum to 100%.

We identified 4 themes (available services, caller characteristics, quitline service provider characteristics, and logistics) and 10 codes (Table 2). Responses did not generally vary by smoking status, age, race, or rural residency. 

**Table 2:** Selected Quotes Illustrating Knowledge and Perceptions of Quitlines, by Theme (n = 4) and Code (n = 10), Study on Perceptions of the US National Tobacco Quitline Among Adolescents^a^ and Adults^b^ (n = 159), 2012–2013^c^

Themes, Codes, and Illustrative Quotes
**Available Services**
**Telephone-based counseling for emotional and practical smoking cessation support**
• Uh, I feel like it would be a mostly like a generic phone call. . . . I mean it could be like a caring person who might try to talk you through quitting. They’d probably . . . give you, like, different ways to try to quit. [Adult nonsmoker]
• Kinda asking . . . why do you think you smoked? Do you realize it’s bad for you? Do you realize if they had, you know, family members, or other people around ’em? But, you know, tell ’em that’s affecting and there is answers, there is ways to get, you know, to stop, and I’m glad you called, you know, this is the first step. [Adult nonsmoker]
• Well, all they would probably do is give you information on how to quit. Probably tell you to go online and look at, read some papers or something and do something else, but they’re not gonna do much for ya. [Interviewer: “Okay and why . . . ?”] Because they’re over the phone, they can’t do anything over the phone for you. [Skepticism about efficacy^d^] [Adult smoker]
**Referrals to in-person counseling**
• They would be setting up appointments to someone help you maybe go to a group for nonsmokers maybe. Because there are smokers meetings maybe, I don’t know. [Young adult nonsmoker]
• [If] they’re an avid smoker, like, just send me to some therapy or something. [Young adult nonsmoker]
• They’d probably have someone pick up and then they’d probably try to refer you, like, ask you where you live, try to refer you to a hospital that puts them on like some program. [Interviewer: “Anything else?”] Rehabilitation, I guess. [Adolescent nonsmoker]
**Tangible smoking cessation resources**
• Maybe it would be someone that could give you references on how to quit. Like, you know, different patches and gum and information. [Young adult nonsmoker]
• I think it’s like they give you like resources as stuff as far as quitting and stuff. [Young adult smoker]
• Refer them to a website. [Adult smoker]
**Caller Characteristics**
**Highly motivated people call**
• Um, I think the people calling the line would be people who might have found out something serious about their health or who might have been, like, the people who smoked a lot and then tried to exercise and then realized they couldn’t do it and realized they needed to, like, change something about them. [Young adult smoker]
• I think people that are at their limit. They’re ready to quit. [Adult nonsmoker]
• Uh, they might need help and nobody else they know could help. [Adolescent nonsmoker]
**Reluctance to call the quitline**
• Personally, probably not. [Interviewer: “And why not?”] Um, I feel like . . . if I really, really wanted to [quit smoking], then I would be able to do it. I haven’t really tried, it hasn’t been too important to me, but I don’t feel like [the addiction is] so serious that I would need a nicotine patch or need a support line like that. [Young adult smoker]
• Um . . . most people usually don’t like to seek help. . . . But around my age, we, uh, start to think about the future . . . [M]aybe someone would call the number, but I honestly don’t think anyone would in my age range. People older, who have been smoking for a while probably would. [Adolescent^d^ smoker]
• I guess maybe after I had several failed attempts that might be a resource. It would probably be third, fourth, fifth down the line, not that it wouldn’t be an option. But, uh, it wouldn’t be the first couple options. [Adult smoker]
**Quitline Service Provider Characteristics**
**Type of service provider**
• Um, I want to say the government or someone. Or maybe like a Red Cross organization or something along those lines. [Young adult smoker]
• I would say it’s probably money from the lawsuits that they sued the tobacco companies with. That they set up programs to help people quit. And this would be, like, one of those programs. [Adult nonsmoker]
**Staffing**
• Well, I’d hope it’d be a person, but it could be a menu that would just try to give you information by you puttin’ in information through your phone. [Adult nonsmoker]
• I would think that it would probably be the moderator for the support group? [Uncertain tone] [Young adult nonsmoker]
• Probably the best people to work there would be ex-smokers. Because they know what kind of stuff people are going through. [Adult smoker]
**Training**
• They could . . . just be random people lookin’ for a job. [Adolescent smoker]
• Um, maybe doctors or, you know, um, psychologists. [Adolescent nonsmoker]
• It’s . . . that first few seconds of, ‘Hi, thank you for calling.’ And you have to teach them that . . . ’cause you’re talking about saving people’s lives, and you’re gonna put on somebody who’s having trouble with his wife, so he brings that. . . . Leave it in the car, pretend you’re happy. You’re saving people’s lives! [Adult nonsmoker]
**Logistics**
**Is there a cost?**
• Probably not like the phone call itself, but through like counseling or the medication it would cost them money. [Adolescent nonsmoker]
• I don’t think there’d be a charge. [Young adult smoker]
**Is the call confidential?**
• I think they would [tell parents] if it has to do with your health. Which, smoking risks can cause various things to go wrong. [Adolescent^d^ nonsmoker]
• Uh, I would hope so. I mean, I guess it’s really not a big deal. People, if you smoke, it’s kinda hard to hide it. Your clothes smell like it and everything else. [Young adult nonsmoker]
• It seems like nowadays there’s a lot of, um, recordings, you know. You call something and ‘this call may be recorded for quality purposes’ and stuff like that, you know. [Adult smoker]

a Adolescents were aged 13 to 17 years.

b Adults were classified as young adults (aged 18–24 y) and adults (aged ≥25 y).

c In-person interviews were conducted in private rooms at 36 recruitment locations in 6 states: Florida, Iowa, Illinois, Kansas, Missouri, and New York.

d A perspective unique among members of the participant’s subgroup.

### Available services

Participants were generally knowledgeable about what the quitline was and what services it provides. Most participants recognized the quitline number as a toll-free helpline and some compared it to other crisis hotlines (eg, suicide). Adolescents seemed to have less specific knowledge than adults; many adolescents mentioned that the quitlines could “help” but did not elaborate further.

Participants identified 3 broad categories of services provided (Table 2). The largest category included telephone-based counseling that provided emotional and practical support for quitting smoking. Terms describing this category included “advice,” “guidance,” “support,” “encouragement,” “tips,” and “strategies.” A small number of participants noted that quitline counselors might educate smokers about the harms of cigarette smoking. Others questioned whether a telephone-based quitline could really help people quit.

Another category of services included referrals to in-person counseling. This category included being referred to local “groups,” “therapy,” smoking cessation “classes,” or “a doctor.” Unlike adults, adolescents mentioned residential treatment programs and “rehab.” Some people viewed the quitline as providing only referrals, without realizing that quitlines offer telephone-based counseling and other services.

The third category included tangible smoking cessation resources such as nicotine replacement “patches” and “gum” as well as pharmacotherapeutic “medicine” or “drugs.” Mailed or online reading materials were also mentioned. No participants reported awareness of text messaging or Web-based programs.

### Caller characteristics

Discussion about who would call the quitline was included in 2 categories (Table 2). The content of the discussion did not vary between smokers and nonsmokers, who were asked to consider why “someone” might or might not call.

First, participants overwhelmingly indicated that people who call quitlines are highly motivated — even desperate — to quit, and are sometimes prompted to call in response to a health problem or crisis. Participants believed that callers would be long-time, heavy smokers who were highly addicted to nicotine, could admit that they needed help quitting, and may not have the social support or skills to quit on their own.

The second category highlighted reluctance to seek help using the quitline (Table 2). With a few exceptions, there was general agreement that smokers would not call the quitline. Some adolescents thought people their age “probably don’t care.” Many adults said they could quit on their own without help, were not highly addicted to nicotine, or already had enough knowledge about cessation strategies and pharmacologic therapies. Several statements suggested that calling a quitline might be stigmatizing. For example, 1 adult mentioned being “too proud and stubborn” to call; an adolescent indicated that people “don’t usually like to seek help.” Although several smokers indicated that they might consider calling when they were ready to quit, they would only do so if they had trouble quitting on their own, if they had health problems, or if family pressured them to do so. The few smokers who said they would call a quitline in the near future tended to be adults who had tried and failed to quit previously and were open to new suggestions or wanted free NRT.

### Quitline service provider characteristics

Perceptions of quitline service provider characteristics were classified into 3 broad categories: sponsors, staffing, and counselor training (Table 2). Most participants believed that quitline sponsors were large government agencies (Food and Drug Administration, US Department of Agriculture, US Department of Health and Human Services, US Surgeon General’s office) or nonprofit organizations (American Cancer Society, Red Cross, anti-tobacco advocacy groups). Universities, health care organizations, and doctors were also mentioned. Many participants suggested that quitlines were funded by tobacco taxes or settlement funds, and a small number mentioned that tobacco companies fund the quitlines.

Participants anticipated a variety of staffing situations, including having calls answered by an automatic voice response unit. Most participants said callers would speak with a live person such as a counselor, volunteer, or paid employee. Many participants specified that quitline staff would be current or former smokers or be personally affected by smoking. Some people believed former smokers were the ideal quit coaches, because they could better identify with and assist callers.

Perceptions of counselor training varied widely, from professionals to “psychology students” to “someone who needs a job.” This variability was further demonstrated by job titles participants mentioned, such as “receptionist,” “resource specialist,” “interviewer,” and “therapist.” One smoker described the importance of training the quitline staff to appreciate the importance of their job in helping “save lives” and avoid insensitivity or letting their own stressors affect their work. Some participants echoed this sentiment by envisioning counselors as having a “calm voice” and being “not rude,” “caring,” and “patient.”

### Logistics

There was overwhelming agreement that an initial call to the quitline would be free. Most of the responses suggested that participants had not thought about costs previously. Feelings of uncertainty about the costs were reflected in words such as “should be,” “probably,” “hope so,” and “think so.” Many participants distinguished a free initial call from additional calls or services that may require payment (counseling, pharmaceuticals). Some adults reflected that the services covered currently by insurance or government funding may not be covered in the future.

Participants generally agreed that quitline services would be confidential, but some participants voiced concerns about the limits to confidentiality. Discussions of confidentiality were also couched in terms that connoted uncertainty (“hope so”), and specific concerns varied by age. One adolescent stated that parents would be notified. Some adults indicated that confidentiality would not be an important concern because smoking “is not a big deal” and it is difficult to hide your smoking status when your clothes smell. However, 1 older adult voiced a concern about being listed as a smoker in a registry and questioned whether that could affect health insurance rates. Some adults also mentioned that the quitline would use personal information for research.

## Discussion

The results of this study indicate that people are generally familiar with the purpose of quitlines, but they have different perceptions of services provided and their operations. Participants overwhelmingly agreed that quitlines are (or should be) free and confidential services that “help” smokers who are highly motivated to quit smoking. However, perceptions of the type of person who calls the quitline were fairly negative, and calling a quitline was largely seen as a strategy of last resort for “desperate” people. Smokers also varied in their perceptions of the training of the quitline coaches, but several emphasized that the ideal coach should be caring, attentive, and a former smoker. Smokers’ reluctance to call the quitline in the near future, willingness to consider calling only if they could not quit on their own, and concerns about the demeanor of quitline coaches are consistent with research indicating that perceptions about not being strongly addicted to nicotine, about having outside support, and about the stigma of using quitlines are barriers to quitline use (16,17).

Many participants expressed nuanced views about the limits of confidentiality and free services. Unlike research that identified concerns about disclosing personal information to a stranger (quitline coach) (17), most of our participants were unconcerned about disclosing their smoking status. However, they did express concerns about other aspects of confidentiality.

Several aspects of the quitlines were misunderstood. Some people thought that quitlines referred people elsewhere for help, including in-person support groups or “rehab” (adolescents only). No participants reported awareness of text messaging or Web-based programs, nor did participants discuss calling the quitline in anticipation of a planned quit attempt, which is the core service of quitlines.

With the few exceptions noted above, the results were relatively uniform across sociodemographic and smoking status subgroups. This uniformity probably results from the straightforward goals of the quitline portion of the interviews: to assess basic knowledge and perceptions of the quitline and its functions. The general ideas conveyed by participants (eg, “helplines” are often free and confidential) were similar across population subgroups. Nevertheless, efforts to promote quitlines within a specific population subgroup would benefit from intensive formative research on sociocultural facilitators and barriers to calling a quitline. It is also important for nationwide marketing campaigns to depict quitline callers and counselors who are similar racially, ethnically, and socially to the target audience.

Our results suggest that future tobacco cessation marketing campaigns should focus on destigmatizing help seeking and should emphasize that professional resources can supplement informal support systems. Advertisements should clarify that quitlines can be used in anticipation of a planned quit attempt; are confidential; offer free and efficacious telephone counseling, text messaging, and Web-based programs; and provide free NRT for eligible smokers. Advertisements should also provide information about the qualifications of quitline coaches and emphasize their interpersonal skills.

Our qualitative study with a convenience sample of adolescents and adults was not intended to estimate the population prevalence of perceptions of tobacco quitlines or to generalize to the US population. Nevertheless, this sociodemographically and geographically diverse sample provides more confidence that our findings are not specific to a particular state or media campaign (3,4,7,16,17), nor are they unique to a particular sociodemographic or smoking status subgroup. Thus, they provide some insight into how a wide variety of people in the United States thinks about quitlines at a general level, including nonsmokers who may recommend quitlines to family or friends who smoke (26).

These data were collected during the “Tips from Former Smokers” nationwide tobacco control campaign (26,30). Our study did not address this campaign, but campaigns like it might benefit from emphasizing the nature and quality of quitline services. The interviews included only a few questions about tobacco quitlines, and participants’ personal experiences with quitlines were limited. The brevity of the interviews precluded exploration of cultural norms and barriers to using quitlines (eg, help seeking), which future research should address. Furthermore, adolescents were more sociodemographically diverse than were adults, which makes direct comparisons across age by sociodemographic characteristics problematic. Views about the quitlines in general, about the government’s role in the quitlines, and about funding through tobacco taxes or settlement funds may have been more salient because participants were interviewed in the context of a study about graphic warning labels.

Tobacco quitlines are highly effective, but their public health impact is attenuated by limited use. Our study highlights several perceptions and misperceptions that may influence smokers’ decisions about calling a quitline. Marketing resources should be directed toward clarifying information and reducing the stigma associated with key topic areas, including the misperception that quitlines are a resource of last resort. Such clarification and destigmatization may increase use of these services, and consequently, reduce tobacco use, improve public health, and reduce tobacco-related health disparities.
